# Radical Cure of *Plasmodium vivax* With Primaquine and CYP2D6 Polymorphism in Southeast Asia

**DOI:** 10.1093/cid/ciae483

**Published:** 2025-03-27

**Authors:** J Kevin Baird

**Affiliations:** Oxford University Clinical Research Unit, Faculty of Medicine Universitas Indonesia, Jakarta, Indonesia; Centre for Tropical Medicine and Global Health, Nuffield Department of Medicine, University of Oxford, Oxford, United Kingdom

**Keywords:** latent malaria, chemotherapy, primaquine, CYP2D6

**(See the Major Article by Zeng et al on pages 379–86.)**

The malaria caused by infection with *Plasmodium vivax* very often comes with persisting latency even after successful therapy of the acute attack with blood schizontocidal therapy [1]. Dormant hepatic hypnozoites account for latency and require specific treatment with either tafenoquine or primaquine, both 8-aminoquinoline drugs. Absent such therapy, subsequent clinical attacks, called relapses, may occur without the further involvement of a mosquito vector. The administration of both blood schizontocidal and hypnozoitocidal therapies is commonly called radical cure. In practice, radical cure is complicated by 8-aminoquinoline pharmacogenetic factors of toxicity and metabolism, along with pharmacodynamic interactions of the 2 classes of drugs applied [2].

Although single-dose tafenoquine combined with chloroquine for the radical cure of *P vivax* infection has become available [3], daily primaquine for 7 to 14 days combined with chloroquine or an artemisinin-combined therapy remains the most widely applied radical treatment [4]. Whether tafenoquine or primaquine is applied, the treatment is potentially dangerous without screening for glucose-6-phosphate dehydrogenase (G6PD) deficiency, a highly prevalent (on average, 8%) genetic abnormality among people residing in malaria-endemic zones [5]. In the specific instance of tafenoquine combined with dihydroartemisinin-piperaquine rather than the approved partner chloroquine in radical cure, poor efficacy against latency has been observed [6]. This effectively prohibits the use of tafenoquine where *P vivax* is resistant to chloroquine and no longer usable, as occurs in Indonesia and the Western Pacific [7]. Primaquine, on the other hand, appears to be efficacious with either blood schizontocidal class in patients who do not exhibit cytochrome P450 2D6 (CYP2D6) natural polymorphisms of impaired activity [8]. In those who do, a high risk of primaquine therapeutic failure against latency has been reported [9, 10]. Primaquine is a prodrug requiring activation by CYP2D6 to kill latent hypnozoites [11], whereas tafenoquine does not appear to be CYP2D6-dependent despite also likely requiring activation (by as yet unknown means) [12].

Historically, 8-aminoquinoline chemotherapeutic strategies have applied relatively minimal doses in order to mitigate the potential harm done by inadvertent dosing of patients with G6PD deficiency. The vulnerability of *P vivax* hypnozoites to these drugs has been highly variable among geographic strains of the parasite; Korean strains are effectively treated with a fraction of the dose required against the tropical Chesson strain, for example [13]. The net effect of this natural variability, combined with conservative strategies of 8-aminoquinoline therapies of poorly understood pharmacogenetic metabolism and drug–drug pharmacodynamics in radical cure, is a great deal of uncertainty in both safety and efficacy of radical cure in a global sense. Despite the nearly 100 years of scientific radical cure of *P vivax* malaria using 8-aminoquinolines, even today there is no consensus among experts as to broadly safe and effective 8-aminoquinoline dosing strategies, be it with primaquine or tafenoquine [14, 15]. This imposes serious difficulties for national malaria-control programs that must make specific recommendations for implementation of the practice of radical cure in patients infected by *P vivax*.

In this issue of *Clinical Infectious Diseases*, Zeng et al [16] offer evidence clarifying at least 1 issue of radical cure strategy in the Greater Mekong Subregion (GMS) in Southeast Asia. Among several hundred patients of the Kachin ethnic minority and naturally infected by *P vivax* of that region, a relatively high proportion of them (75%) had CYP2D6 genotypes of predicted impaired metabolism (activity score <1.25). Most important, those same patients had a more than 6-fold higher risk of *P vivax* recurrence during the year following radical cure with chloroquine and low-dose primaquine (total dose of 3.5 mg/kg rather than 7.0 mg/kg over 14 days). The observed estimated efficacy against relapse in this population was no greater than 75%. A high prevalence of impaired CYP2D6 polymorphisms resulted in relatively poor efficacy of the low-dose primaquine regimen. In this area of the GMS involving 1 ethnic group, the lower dose of primaquine for radical cure too often resulted in therapeutic failure against latency. A relatively high frequency of impaired CYP2D6 alleles appears to have been directly involved in that outcome. Although impaired metabolism by CYP2D6 may also be relatively common among broader Southeast Asian populations, the null metabolizing phenotype (where both alleles are wholly disabled) is uniformly relatively rare [17]. The problem of impaired primaquine metabolism may thus be mitigated or wholly addressed simply by applying the higher-dose primaquine regimen (7.0-mg/kg vs 3.5-mg/kg total dose).

Over the past 2 decades, substantial resources and effort have been devoted to the elimination of artemisinin-resistant *Plasmodium falciparum* in the GMS [18]. While tremendous gains were made against that species, none occurred with *P vivax*, very probably due, in part, to poor uptake of radical cure in practice due to fear of toxicity in G6PD-deficient patients [19]. While prudent precaution with 8-aminoquinoline therapies is always indicated, with or without G6PD screening [20], safely using the higher-dose regimen of primaquine with such screening would perhaps overcome the otherwise serious problem of therapeutic failure linked to high frequencies of impaired CYP2D6 alleles in this region. The report from Zeng et al [16] offers evidence inferring a need for national malaria-control programs in the GMS to adopt and adapt to higher-dose primaquine for radical cure of *P vivax* malaria.
